# Colorectal cancer community engagement: a qualitative exploration of American Indian voices from North Dakota

**DOI:** 10.1186/s12885-021-09119-2

**Published:** 2022-02-09

**Authors:** Nicole Redvers, Mia Wilkinson, Courtney Fischer

**Affiliations:** 1 Department of Family & Community Medicine, University of North Dakota School of Medicine & Health Sciences, ND Grand Forks, USA; 2 Department of Indigenous Health, University of North Dakota School of Medicine & Health Sciences, ND Grand Forks, USA

**Keywords:** American Indian, Colorectal cancer, Community engagement, Cancer prevention, Cancer education, North Dakota

## Abstract

**Background:**

American Indians (AI) in North Dakota present with higher rates of advanced-stage disease for screening detectable colorectal cancers and have lower overall baseline colorectal cancer screening rates than non-AIs. We sought to identify the perceived barriers and facilitators for the engagement with colorectal cancer prevention within North Dakota tribal communities.

**Methods:**

Twelve semi-structured interviews were carried out across four tribal reservation communities in the state of North Dakota with American Indian adults between the ages of 30 and 75 years. We utilized purposive sampling to ensure maximum variation in age, sex, and tribal community until data saturation was achieved. The interviews were transcribed, and thematic analysis was carried out to identify consistent themes rooted within the data. Ethical approval was gained for this project from all relevant institutional review boards.

**Results:**

Four main themes were identified as barriers for the engagement with colorectal cancer prevention, including: colorectal cancer screening barriers, focused on other health problems, lack of colorectal cancer tailored health promotion, and socio-cultural factors affecting colorectal cancer prevention. Three main themes were identified as facilitators for the engagement with colorectal cancer prevention, including: reasons for getting colorectal cancer screening, role of culture, and getting out into the community.

**Conclusion:**

There is need for more community-rooted, strengths-based approaches to colorectal cancer prevention activities in AI communities in North Dakota. Socio-cultural factors, such as the use of storytelling, and the use of traditional knowledge have been demonstrated to be an important element of consideration for colorectal cancer tribal community engagement and prevention planning in the state.

**Supplementary Information:**

The online version contains supplementary material available at 10.1186/s12885-021-09119-2.

## Background: reflection

American Indian and Alaska Natives (AI/AN) in the United States (US) are resilient and diverse in their languages, cultural practices, land bases, and governance practices. Therefore, the frequent cultural homogenization of AI/AN communities across the US continues to perpetuate harmful stereotypes and increases the likelihood of poor health outcomes [1]. Broad, mainstream approaches within health education and promotion for AI/AN do not consider the unique strengths and challenges within each respective AI/AN community and assumes that what works in one AI/AN community will automatically work in another AI/AN community [2–4]. With this, there is great need in AI/AN research to differentiate between what we are calling meta-level scholarship (i.e., that which seeks to find common overarching drivers of behavior within health prevention activities due to *collective experiences* [5]), and implementation scholarship (i.e., that which seeks to develop and apply health prevention activities on the ground in communities [2]). Better cross-disciplinary efforts are needed to ensure research is relevant, applicable, responsive, and driven by the needs of local AI/AN communities within implementation processes with all scholarship endeavors needing to be firmly aware of the cultural uniqueness between regions [6].

From the meta-level, AI/AN communities in the US do share the collective experience of colonization, which has led to cumulative and ongoing impacts on land quality and access, traditional food and cultural practices, historical trauma, racism, white supremacy, and the consequent extreme health disparities in conditions such as diabetes, heart disease, and cancer [7–10]. It is through the ongoing process and experience of colonization that the viewpoints and engagement with cancer prevention activities may converge to overarching points of either contention or facilitation within certain regions. Through the shared historical power differentials potentially perceived or outwardly displayed from clinicians to patients [11], the access or lack thereof to culturally safe care [12], worldview differences between biomedical scientific approaches and traditional healing approaches [13–15], and the overall lack of knowledge within health systems broadly on historical trauma [16, 17], this research was interested in *exploring for the possibility of shared elements within colorectal cancer engagement across a sub-set of tribal communities within the state of North Dakota in the US*. This process was not meant to minimize the culturally distinct elements between tribal communities in North Dakota, but to elevate and strengthen the discussion on potential shared *structural elements* that can exist within a region due to the collective experience of colonization.

American Indians (AI) generally present with higher rates of advanced-stage disease for screening detectable cancers, have a lower level of basic cancer screening knowledge, and often have more negative attitudes about Western cancer treatment than non-AI patients [18–20]. In North Dakota specifically, the prevalence of those between the ages of 50 and 75 years as of 2018 who have had a colonoscopy in the past 10 years was substantially lower for AIs compared to Whites (47.1% vs. 64.7% [21]). AIs also had a higher age-adjusted colorectal cancer mortality rate than Whites between 2014 and 2018 in the state (17.6 vs 14 per 100,000 [22]). Due to the last known research from 2011 demonstrating that one-third of screening eligible AIs in the state of South Dakota (adjacent to North Dakota) were not planning to receive cancer screening [23], we specifically sought to identify the currently perceived barriers and facilitators for the engagement with colorectal cancer prevention within North Dakota tribal communities. We also wanted to identify community-derived solutions for improving colorectal cancer preventative measures within the region. Many great local initiatives have been established to improve the uptake of colorectal cancer screening in North Dakota tribal communities. These local initiatives should be commended for their hard work. This current research, therefore, seeks to build off the local initiatives’ great momentum to not only give voice to the initiatives themselves as reflected through community members, but also to be honest where efforts may need additional elements of consideration or prioritization based on the collected data.

### Relationships and study context

Given the self-location of the researchers as well as the community voices we sought to elevate, it is important for us to ground ourselves and our readers in this work [24]. The principal investigator (N.R) is a member of the Deninu K’ue First Nation and has been working in Indigenous health as either a clinician or researcher for over 12 years in Canada and the US. The second author (M.W.) is a member of the Three Affiliated Tribes (the Mandan, Hidatsa, Arikara Nation) from North Dakota in the US. The third author (C.F.) is Cheyenne River Lakota originally from Eagle Butte, South Dakota, and is an enrolled member of the Cheyenne River Sioux Tribe. All the authors (N.R., M.W., C.F.) position themselves with the intent to improve health within AI communities as part of the reciprocal relationship and actioned responsibility honored within Indigenous communities and Indigenous research methodologies [25].

## Methods: listening to American Indian voices

### Research aim and questions

Our aim for this research was to explore for the possibility of shared elements within colorectal cancer engagement across a sub-set of tribal communities within the state of North Dakota in the US. We specifically sought to identify the currently perceived barriers and facilitators for the engagement with colorectal cancer prevention within North Dakota tribal communities (question #1). We also wanted to identify community-derived solutions for improving colorectal cancer preventative measures within the region (question #2).

### Overall design

We designed a qualitative research study to better ensure the ability to listen and engage directly with participant voices. It is often not culturally appropriate to alter someone’s words in Indigenous research settings [15], so by having a study that utilized interview techniques as the data collection method, we were able to ensure our participants’ voices were heard, honored, and respected. The honoring of voices is considered standard practice in many Indigenous research methodologies [26], and by actioning responsible reciprocity [25], the research data is better able to reflect the local community experience of colorectal cancer engagement.

We prioritized a decolonized research paradigm utilizing elements of decolonial theory and Indigenization, which involves a “collaborative process of naturalizing Indigenous intent, interactions, and processes and making them evident to transform spaces, places, and hearts” ([27], p.7). This prioritization allowed us to focus our intent on positive impact for the communities. Utilizing an Indigenous methodological orientation does not preclude the ability to carry out a rigorous process of research, and we additionally followed standards for reporting qualitative research (SRQR [28]) in this study.

### Recruitment

Ethical approval was attained for this project from the University of North Dakota Institutional Review Board (IRB-201909-066), the Sitting Bull College Institutional Review Board (#SBC219), and the Turtle Mountain Band of Chippewa Research Review Board (#113). This manuscript was submitted to the relevant tribal review board before publication following conditions outlined in the ethical approval process.

All participants gave informed consent and were offered a $25 gift card as an honoring process for their participation. Standard Western research incentive guidelines are normally focused on reimbursement or incentivization for participation in the research process [29]; however, many Indigenous traditional protocols instead have a reciprocal responsibility innate in the participation process, which means an honoring of the time given through gift giving [30]. Indigenous traditional protocols are like guidelines for living life in a good way, and they are based on the need for respectful balance in all ways of living (i.e., if I gain something from you such as knowledge or stories, it is important to then provide a gift or offering back to keep things in balance).

Potential participants were recruited through established local networks of the authors utilizing purposive sampling to ensure maximum variation in age (> 18 years), sex, and tribal community until data saturation was achieved [31]. Only self-identifying American Indians living within North Dakota tribal communities were included in the sample. Snowball sampling was additionally used in a few instances to broaden the participant base as needed [32].

### Interview data collection

Semi-structured interviews were carried out virtually in English between March 11, 2021, and May 6, 2021, due to pandemic restrictions precluding the possibilities for in-person interviews. Video conferencing software was utilized with two authors (M.W., C. F.) trained by the principal investigator (N.R.) in interview methodology prior to carrying out the interviews. Each of the interviews were audio-recorded and supplemented with memo taking throughout to ensure ongoing reflection of the data being collected. Participants were made aware of the audio recording and were provided an opportunity to stop their participation if they did not feel comfortable.

An interview guide was developed after a review of the literature, as well as an internal review of relevant local cultural elements to ensure appropriateness of the questions. Participants were asked open-ended questions following the developed discussion guide (see *Online Resource 1*) while allowing for flexibility to explore and probe areas that emerged as part of the data collection effort. Participants were queried on what they thought their communities’ strengths and challenges were regarding cancer prevention, preferred learning methods for colorectal cancer, and any cultural factors that may impact colorectal cancer prevention.

### Data analysis

Once the interviews and reflective memos were completed, the interviews were transcribed verbatim by the research team (N.R., M.W., C.F.) and uploaded into NVivo (Release 1.3) qualitative software for further analysis. Interviews were analyzed concurrently with data collection to help inform subsequent interviews and probe into any relevant findings emerging from the data. Thematic analysis was carried out following Braun and Clarke’s (2006, 2020) six phases [33, 34]. Phases 2 (systematic data coding), 3 (generating initial themes from coded and collated data), and 5 (refining, defining, and naming themes) of Braun and Clarke’s (2006, 2020) thematic analysis process [33, 34] were separated into isolated folders in the qualitative software with ongoing memo taking throughout the process to ensure an audit trail of the data at each stage of the process.

Two authors were involved in the coding process (N.R., C.F), with the third author brought in for discussion as needed (M.W.) to further define and clarify emerging themes. Coders constantly referred back to the original transcripts when reviewing narratives to ensure an iterative process that facilitated a clear signal when data saturation had been reached [31]. Data saturation was determined to be reached when no new themes emerged from the data analysis process [31]. For each of the relevant themes, memorable participant responses were organized into sections for the purpose of reporting.

## Results: learning from American Indian voices

*“… when you talk about cancer, it's a two-edged sword. You want to know about it, and you don't want to know about it. Stay away from me, out of sight, out of mind. Stay away from me. If I don't look at you, you don't see me, if I don't talk about you, you don't come towards me …”* (ID 426)

Twelve semi-structured interviews were carried out across four tribal reservation communities in the state of North Dakota. Participants were self-identifying American Indian (AI) adults between the ages of 30 and 75 years (*N* = 7 female identifying, *N* = 5 male identifying). We platformed our analysis within a vision of learning from our participants and identified synergies across the tribal communities. Therefore, the results presented represent themes that were cross-cutting across the majority of the participants.

Four main themes were identified as barriers for the engagement with colorectal cancer prevention (see Table 1), including: colorectal cancer screening barriers, focused on other health problems, lack of colorectal cancer tailored health promotion, and socio-cultural factors affecting colorectal cancer prevention. Three main themes were identified as facilitators for the engagement with colorectal cancer prevention (see Table 1), including: reasons for getting colorectal cancer screening, role of culture, and getting out into the community.

**Table 1:** Themes identified as the main barriers and facilitators for the engagement with colorectal cancer prevention

Themes	Categories
**Barriers:**	
*Colorectal cancer screening barriers*	-Living in a rural area -Lack of local healthcare capacity -Transportation barriers
	-The screening procedures are not fun
	-Unique barriers for American Indian men
*Focused on other health problems*	-N/A
*Lack of colorectal cancer tailored health promotion*	-Access to healthy foods and activities is limited
	-Not enough awareness and education
*Socio-cultural factors*	-Avoiding healthcare until symptoms worsen -Fear of cancer being found
	-Colorectal cancer not talked about -Embarrassment due to private nature of body part
	-Mistrust of Western healthcare systems
**Facilitators:**	
*Reasons for getting colorectal cancer screening*	-Having a role model or community spokesperson
	-Importance of family or health provider pressure
	-Worsening of symptoms
*Role of culture*	-Use of storytelling
	-Use of traditional knowledge, ceremony, and prayer
*Getting out into the community*	-Community programming and events
	-Importance of visual education materials

### Barriers

It is important to note that the four main themes that were identified and presented here as barriers for the engagement with colorectal cancer prevention were often overlapping. It was extremely rare to have only one barrier noted by a participant, with many cases having multiple interrelating barriers noted that affected each other in ways that seemed to amplify the perception of the barriers (e.g., mistrust of Western healthcare systems potentially impacting the avoidance of healthcare until symptoms worsen).

#### Colorectal cancer screening barriers

There were three main cross-cutting categories identified as barriers for colorectal cancer screening itself, including: (1) *living in a rural area* (which included two sub-categories of a lack of local healthcare capacity and transportation barriers), (2) *the screening procedures are not fun*, and (3) *unique barriers for AI men*.

Due to *living in a rural area*, most of the participants were keenly aware of the overstretched healthcare systems in their communities, citing a lack of capacity for being able to get cancer care locally, and the lack of stable healthcare providers.

I would say nurses and primary care providers are the ones who are working tirelessly to bring awareness [about cancer], and they do a good job, but, they just have so much to do and so many people to serve. I'm sure their ratios here are probably much higher than other places … they're just stretched very thin … they're probably always getting pulled to do more urgent things. (ID 423)

As having a lack of local healthcare capacity in rural areas means that patients will often have to travel to receive a colonoscopy, transportation barriers were repeated throughout the interviews.

… generally, our Elders don't go to nursing homes and they're dependent upon the family to transport them. Whether we have to drive an hour to a [community] or three hours over to [community]. And so, many of the Elders are not wealthy enough to own and buy their own car and then have somebody else drive them. (ID 424)

I don't know any Indians that want to go drive seventy-five miles and have a colonoscopy. (ID 424)

This transportation barrier in rural areas was noted not only in terms of access to transportation but also the travel time requiring extended time off work, which was felt to be not feasible for many.

… when we are willing to take that drive, it's like I need to take that drive like something wrong is happening. So, I need to like take a whole day off work to go to the hospital or to the clinic or whatever. So, yeah … that's an access to services type of thing. (ID 423)

There were some notable and successful programs that had been set up to address this transportation issue noted by some of the participants, including transportation arrangements being organized and paid for within the community to reduce this barrier; however, some participants were unsure if this existed in their communities. Compounding the travel barrier was the perception that the *colorectal cancer screening procedure was not fun*, and travel exacerbated this reality.

But the prep is a nightmare. And for us people, like I had to go to [community] the night before and pay for a hotel because, you know … I wasn't about to have to drive from here to there at any time, you know, because it's not like there's restrooms every 10 feet and you literally need to be right next to the bathroom and, you know … and I can't imagine people who have to do that all night and then catch the bus at 4:00 in the morning to go to [community]. I mean, what a nightmare. And I know of somebody who had to cancel theirs because they had an accident trying to get there, and that's a nightmare. So, I think they could do a lot to make it easier … and again, it's just us being so rural. (ID 653)

AI men living in tribal communities were identified as a demographic in need of more support for colorectal cancer screening (i.e., *unique barriers for AI men*).

I mean, to me, it seems like it's harder for ... kind of like the older guys to go in, I mean, it's like it's just hard to learn to go into hospital. (ID 427)

Many interviews described the challenge with men only accessing medical care unless there was something very wrong going on, and that men had a harder time than women talking about colorectal cancer screening in general due to its perceived private nature.

I believe men don't go get screenings as much as women. I think there should be more of an opportunity for men, because men are I think … I just think my personal belief is men are more private. They don't like to ... to do stuff like that, they prolong it until their illness has already come upon them. They don't do preventative I don't think as much. (ID 655)

In some cases, women felt they had a hard time approaching men with this conversation due to perceived cultural barriers.

… I would just say like a respect thing that you would have for opposite genders or Elder populations and like I would talk to my grandma about it, but I probably wouldn't have talked to my grandpa when he was here about it, you know what I mean? Like, I'd probably tell my grandma to talk to my grandpa and then in trying to tell people to tell people I probably would get lost anyways. But I think it's just because it's an invasive procedure. (ID 423)

The colorectal cancer screening barriers mentioned throughout this theme often intertwined with each other as there was often more than one barrier present for an individual. For example, men often had their own personal comfort barriers to accessing screening, which was platformed on a lack of local health capacity, the need for travel, and the procedure being perceived as outside of their comfort zone. These types of multi-layered and interconnecting barriers were present in many of the interviews as previously noted.

#### Focused on other health problems

One of the other barriers with the engagement of colorectal cancer prevention was the often-perceived focus on other types of health problems that affect AI communities. Many of the interviewees noted awareness of clear community programs and education for breast cancer and even cervical cancer; however, were not able to talk with the same clarity about colorectal cancer.

Breast cancer and pap smears, you know, that's all we're taught throughout all of this and then I think if we would have had … maybe more experience or more learning, and we're taught more about symptoms of the different kinds of cancers, maybe we would listen to our bodies better. (ID 652)

Diabetes was consistently mentioned as the most dominant health problem within the tribal communities. Many felt that the diabetes conversation outweighed that of other conditions like colorectal cancer or even other cancers.

I just think that cancer is a tough topic and I feel like, honestly, I think that cancer gets outweighed by diabetes within the Native American population, like even within myself. (ID 423)

It's just growing up in our community … is predominantly Native American. And you know, we suffer … our biggest almost expectation is to get diabetes. (ID 426)

Participants noted that they felt generally educated on diabetes and were also familiar with various initiatives happening within the community for diabetes prevention; however, they were not able to talk with the same clarity about colorectal cancer.

#### Lack of colorectal cancer tailored health promotion

There were two main categories under this theme including, (1) not enough awareness and education, and (2) access to healthy foods and activities is limited.

The noted lack of clarity in participants on colorectal cancer initiatives stemmed from what many felt was *not enough awareness and education* surrounding the topic was reaching them. There was a sense that existing education was not creating enough awareness about the ability community members had to actually prevent cancer.

… it's just become normal for us to live in stressful environments and it's normal for us to have high rates of cancer or high rates of diabetes and high rates of obesity. And, it's normal for us to think that probably one day we're going to have one of them, and that's just going to be it. Like we are not aware, like some of our population is not aware that we can change that narrative for ourselves. (ID 423)

I think there should be more outreach and more understanding at all levels, from tribal governance to Indian health service, down to our social society, especially in our schools, our colleges. There's kind of not enough. (ID 425)

Participants also felt as though their ability to engage in colorectal cancer prevention was hindered by the *lack of access to healthy foods and activities* in their communities.

… there's a lot of talk about eating healthy foods and things like that, and then my mind goes to the weakness part where we don't have access to that … like we are taught about how to prevent cancer in general, but it's hard to, hard to do because just of access to good food. (ID 652)

I'm going to go shopping now and I have, you know, vegetables and apples and oranges and fruit on my list and those are expensive. It's a lot cheaper to buy a bag of potato chips for a family to fill up the kids than it is to buy fruit and vegetables. (ID 428)

Participants noted that this lack of access to healthy foods and activities in their communities was not only a barrier for colorectal cancer prevention but was also a barrier for preventing the many other health conditions that exist in the community, such as other cancers and diabetes.

#### Socio-cultural factors

There were three main categories under the theme of socio-cultural factors considered to be barriers in the engagement with colorectal prevention. These three categories were: (1) *avoiding healthcare until symptoms worsen*, (2) *colorectal cancer not talked about*, and having a (3) *mistrust of Western healthcare systems*.

*Avoiding healthcare until symptoms worsen* was seen to be, “… because a screening is preventative and we're only going when things are like hitting the fan or when things are extremely urgent, and we need to be like in the emergency room” (ID 423). The delay in accessing healthcare services was multi-factorial, including many of the already presented barriers; however, there appeared to be a culture of waiting that was seen to be prevalent in the communities.

… and generally, they all found out way too late, because I think a lot of them did not get screened, did not go in for whatever reason … and they finally go in when they get severe pain, you know, in the colon or maybe the cancer or tumors pushing on an ovary or in another organ and then having referred pain. And they finally go in and then, boom, they find this out. (ID 424)

… and so the culture, the culture definitely has to shift and hopefully we're working towards that. But right now, the normal is you don't need to go in. You don't … you're not going to go in until you're hurting, unfortunately. (ID 423)

Many of the participants added that another factor in avoiding healthcare until symptoms worsened was the fear of cancer actually being found.

… a lot of people don't advocate for themselves, and they get scared. So, then they, you know, instead of getting scared and being like, oh, test me, test me like I did, a lot of people get scared and then avoid the doctor at all costs. And meanwhile … if they actually have cancer, they're just letting it get worse. (ID 653)

I have friends that, that are older, that have cousins, too, that are my age, close to 70 that haven't had their first breast cancer screening. So, a lot of it is fear of going in, they say they don't want to know. (ID 428)

Compounding this fear of cancer was that *colorectal cancer was noted to be not talked about* among community members.

But you know what? It's not something that your friends like … somebody will just say, oh, I'm going for a colonoscopy next week. And, you know, it just doesn't like come up in conversation. (ID 654)

Potentially underpinning colorectal cancer not being talk about was a clear thread of embarrassment due to the private nature of the body part involved in colorectal cancer (i.e., colon and rectum), which was seen to potentially have cultural underpinnings.

The kids will laugh, the adults will be embarrassed because we're just like, easily embarrassed people, I guess, or like we're just so private. And yet that is because it's so invasive, it's probably just not talked about. (ID 423)

… that's how Native American country is. We're very private people, and so, I think that's a big challenge going on. (ID 426)

An additional category that the majority of participants noted as a barrier with colorectal cancer engagement was a *mistrust of the Western healthcare system*.

… I don't think that people trust our healthcare system, which is Indian health services in Indian country here, especially in the Great Plains. (ID 424)

This was thought to be platformed on both historical elements as well as modern-day issues, including the lack of consistent healthcare providers in the rural clinics.

Like, you have to remember that some of the older populations are not going to trust the government because they came from the bottom lines where they were told they wouldn't get relocated and they got relocated and they were kids at that time. (ID 423)

I think there's a lack of trust because you have generations of Indian health service providers where there's no consistency in care. You have a lot of providers coming in now … you don't have consistency. And so, you have a different provider doctor telling your story to over and over again. And so, it can only lead to mistrust. (ID 429)

Participants were clear to note that issues of mistrust were complex, and often spoke about mistrust in general terms as opposed to it solely being an issue with colorectal cancer prevention activities exclusively.

### Facilitators

#### Reasons for getting colorectal cancer screening

There were three main categories describing the reasons for getting colorectal cancer screening, including: (1) *having a role model or community spokesperson*, (2) *importance of family or health provider pressure*, and (3) *worsening of symptoms*. Many participants noted that it was much easier for them to relate to another Native American than it was to other populations, and *having a role model or a local community spokesperson* that they respected would go a long way in convincing them to go get a colorectal cancer screen.

I think Native Americans are kind of more ... how would you say it … just like, I guess just connect easier with somebody from their own community. (ID 655)

I really think that anytime you have a Native person that you can have as a spokesperson, it's a bigger message that comes across. … Something they can relate to. Somebody they know, like we have had a [person that] told a story that his [relative] had colon cancer and she died, and he was encouraging everyone to follow through and to get the screening. (ID 429)

Having either a family member or one’s healthcare provider giving some positive “pressure” (i.e., *importance of family or health provider pressure*) was noted to have a strong impact on an individual’s willingness to go in for a colorectal cancer screen or to change health habits.

I would get it for my family just because … I would like to be around for my grandchildren and to be a part of life and to be able to take them for walks in the parks and things like that, I mean, that would be for me. I mean, that's one reason why I would be willing to change my way of eating or whatever it takes to try to prevent it as best I could and just take care of myself and have checkups on a regular basis, you know, things like that. (ID 427)

Even if they have to get grandma involved. … You know, how grandma is. (ID 656)

From … either the nurse or the physician … to let them know you haven't had this done since this time, I think you’re due for this, I think it needs to be clearly told to people and remind them. I think that's one of the things that should be done on every visit, whether it's just a routine visit or if you're coming, if you're having a flu-like symptom or whatever, just as a reminder, even though that's not what your main purpose is there for just keep reminding them so that they will get the screenings done. (ID 655)

*Worsening of symptoms* was another clear prompter for getting a colorectal cancer screen. One participant noted in this regard that “… you know, you don’t pay attention to those things until you sit in front of a doctor because you have something you can’t get rid of” (ID 426). It was clear from many participants that although worsening of symptoms was a common prompt for wanting to engage with colorectal cancer screening, this also prompted the engagement with other forms of screening and investigations outside of cancer as well.

#### Role of culture

There were two main categories under the role of culture for facilitating engagement with colorectal cancer prevention including: (1) *the use of storytelling*, and (2) *the use of traditional knowledge, ceremony, and praye*r. Many participants felt that *storytelling* was an effective and more accessible method for getting across cancer health information in a culturally respectful way.

A method of learning is Native Americans really like storytellers. So, you can come up with some storytelling ideas. They'll sit and listen to that because they love storytelling. (ID 424)

I think people like to engage more in speakers, listening to other people. … I think it's more like when you're watching, listening to somebody versus reading, it's easier sometimes for people to comprehend it that way. It's kind of like a hands-on more than textbook reading material, because it's then maybe taught in a easier to understand way than medical terms where it's harder for people to comprehend the language. (ID 655)

Storytelling was a part of the greater array of cultural practices that the majority of participants felt were important in the engagement with prevention activities. *Utilizing traditional knowledge, ceremony, and praye*r were felt to be important aspects that brought strength to the community and the creation of a roadmap for healthy behaviors and relationships.

It's when we talk about our culture and our health … it's always like an eco-system level, you know, be healthy, move more, eat better, eat more Indigenous-type foods. Umm, so and it's all true. It's all something that is needed … because it's we’re, umm, we’re unique. (ID 625)

… our people always had some type of root or medicine in their mouth because it was always about prevention … and I think that's always, you know, it's ingrained in our culture is that we, you know, prevention. We take care of ourselves. (ID 656)

So many of our Elders recently are saying, like I was told, to blend both modern medicine and traditional medicine. … She said, you need to do both. You do both. It's OK to do both. (ID 428)

… I think a lot of our ceremonials and our ways of life in our healthy practices with the medicines that we do have is a big part of prevention … because when you think about the power of prayer, it helps with the stress. If you think about the power of food and nutrition, the way we used to eat and how we eat will help with nutrition, the energy, you know, how active we were, and, you know, just building a sweat lodge or building … just living off the land allows you to be active. So, I think our traditional values and ways plays a big part in prevention for cancer. (ID 425)

Some participants were unsure how traditional Indigenous knowledge could work in Western medical settings; however, they none the less acknowledged the importance of their traditional ways for cancer prevention.

#### Getting out into the community

All participants were very clear to emphasize the importance of getting out into the community for facilitating the engagement with colorectal cancer prevention. There were two main categories noted under this theme including: (1) *community programming and events*, and (2) *importance of visual education materials*. Many participants noted the success of many of the breast cancer awareness events (i.e., *community programming and events)* that they felt could be better leveraged for colorectal cancer prevention activities. Participants also noted the success of getting out information in the local casinos, and public cultural events.

… a gathering at pow-wows or stuff like that, I mean, I think you could probably have a booth somehow set up or whatever to help promote it. (ID 427)

… if we could do that together and create big events for communities, we can have bigger strides in the directions that we're all trying to go … like using possibly using like public cultural events, so things that are more like celebrations, not so much like ceremony … but like utilizing public celebrations and social events, they're utilizing social cultural events to provide education. (ID 423)

There were frequent suggestions of offering specific health programming that focused on general prevention like healthy eating and having public health nurses available more widely in the community at large.

… if we can use the moccasin news and get our home health, public health nurses all working together to get out to the patient's house, as well as wholesome events, you know, some incentive and events. (ID 424)

… all those Elders are, I mean, if you go over and see them and say, oh, jeez, maybe, you know, we need to do this, this, this and oh, by the way, we better do a colorectal cancer screening here. Let's sit down and I got a video, I'll show you about it. They show them, they agree to it and then they do it and we get the screen, and we share the results with them and talk to them. And then they get on the phone the next day and call their friends and say, hey, guess what happened to me, you know? Yeah … and that's the moccasin news that all Elders are dying to get on and they all talk to each other and jeez, you should get one, too, you know. And next thing you know, we get calls from Elders telling them, hey, I'd like to have you come over and check this out. (ID 424)

Many specifically noted the impact the roll-in-colon events had in the community and were able to describe the details of the models that were set up in the community.

… they used to have a roll-in-colon, and I think that'll be a good idea to bring that over. … they had a roll-in-colon and it's actually like a big colon that you could walk through and you could see like a shell of you ... people have cancer, like those little samples of like when you walking, you can see little samples of what different things would be. (ID 429)

There were other mentions of programs that “used to be” in the community but had stopped for various reasons.

… at one time they did the evening one where men were new, it was specifically for them to have screenings done. I think that would be beneficial if something like that was once again brought back where you have just a certain time in for screenings. You know, not just a routine thing, something that is you know specific for that. (ID 655)

Many participants mentioned the *importance of visual education materials* being used in the engagement process that were culturally tailored.

… tell me and I forget, show me and I remember, involve me and I understand, and so … it's been my personal experience with other Native Americans that we tend to learn better from visuals … So those are visually learning, and storytelling would be really excellent ways to look at learning about cancer to the Native Americans in a culturally respective manner. (ID 424)

There was a clear acknowledgement that some of the younger generations might prefer media visuals, whereas the older generations still preferred things like brochures and posters around town; however, regardless of age, most people got much of their information from social media platforms like Facebook and enjoyed watching short videos.

I strongly believe it will be through brochures for the older, older people, the older dynamic, and then also on video for the younger, you know, see Facebook or messages through Facebook. (ID 426)

There was repeated note on the importance of developing visuals representing the community as opposed to having outside visual education materials.

I also like to have like Native Americans as part of the pictures. I think they're doing more of that, but traditionally [previously], there was a lot of non-Natives … so when we have posters … we tried to make sure that there is like they're culturally sensitive, like there's Native Americans that may be part of it … (ID 429)

… I've seen some really good ads on TV for smoking cessation, you know, with Indian people and Native people speaking and stuff. (ID 654)

As was noted in the barriers section, there was much overlap between the themes in the facilitators section. The three main themes that were identified as facilitators for the engagement with colorectal cancer prevention (i.e., reasons for getting colon cancer screening, role of culture, and getting out into the community) were again often interrelating with each other. The role of culture dissipated into other themes, for example, through the use of storytelling within the visual materials, to the importance of role models being vehicles for engagement at community events.

## Discussion: engaging with and elevating American Indian voices

This research was interested in exploring for any potential convergent elements within colorectal cancer engagement across a sub-set of tribal communities within the state of North Dakota. Due to the collective experience of colonization, we sought to investigate any shared barriers and facilitators within the region while also being very mindful of the culturally distinct elements between tribal communities. By engaging directly with participants and communities, we made a strong effort to elevate the collective voice by carrying out research within the qualitative realm. We prioritized facilitators and barriers with the engagement of colorectal cancer prevention that crossed the majority of communities, sex, and age. Through the investigation of any relevant facilitators, we were also able to get a sense of existing or recommended community-derived solutions for improving colorectal cancer preventative measures within the region. Some of these community-derived solutions had already been instigated in some communities but not in others.

Historically, tribal communities in North Dakota have had limited research carried out and published regarding colorectal cancer prevention. To date, the health research on colorectal cancer in the region that has been published has often been quantitative in nature [19, 35, 36]. We felt it was important with this current research to explore for potential nuances within the region that can be difficult to pick up with more quantitative research methods. We were able to note upon final reflection that many of the stories we heard from participants mirrored those found in other American Indian-based qualitative studies on this topic in other regions [37–40]. Similarities were also found between our results and that of other cancers such as breast cancer in other AI communities [41]. This similarity finding may mean that other AI communities’ or regions’ colorectal cancer prevention efforts may have applicability in tribal communities in North Dakota [42–44]. Appropriate local adaptations will still likely be needed due to important cultural nuances. For example, even though storytelling was stated to be a potentially effective method of delivering health education across our study and previous studies with AI communities, there are different community protocols regarding how storytelling is carried out. Cultural nuances such as this example did not come out in our research data but are known by the authors as an element of important consideration for the operationalization of the findings (e.g., some stories are to be told only in certain seasons or settings in some tribal communities). So, general attributes such as the ‘importance of storytelling’ are potentially transferable to other community settings from an operationalization standpoint; however, it must be known that there will still be some level of cultural nuances to take note of as exemplified.

The themes of mistrust of Western healthcare, fear of cancer, dislike of the colorectal cancer screening procedure, embarrassment talking about colorectal cancer due to the private nature of the body part, having a focus on other healthcare ailments such as diabetes, and the importance of cultural factors have been identified as being relevant in other AI/AN communities outside of North Dakota [37–40, 45]. These were all elements that came up strongly in our findings, providing a level of synergy with other communities. This could potentially be due to shared historical and ongoing structural elements (e.g., colonization, historical trauma) that continue to affect many tribal communities in the US [17]. Other elements of note in our study such as transportation barriers, lack of healthcare capacity, and unique barriers for men have been highlighted in other contexts as well [40, 45].

Due to the increasingly overlapping and synergistic barriers and facilitators that are apparent in the AI literature on colorectal cancer prevention (including the addition of our regional study), it is possible that an overarching, high-level framework may be developed for the engagement with colorectal cancer prevention. A framework could be created with the intent to allow flexibility on the local level due to cultural variances, but also help to identify future research needs and operational gaps within a respective region to better inform programming. For communities with limited resources that are wanting to begin preventative programming, having a general framework may also give them a clearer road map leading to quicker operationalization. With this, the development of a framework may also help communities learn from the lessons and successes of other communities without the need for direct partnerships. This framework should not only take into consideration the individual elements that have been identified in our research and in comparative research within AI communities (this has been done to a substantial extent already), but could additionally consider *system level drivers and approaches* that are very rarely emphasized in standard cancer prevention education planning currently.

For example, mistrust of Western medical care is a large barrier that not only affects the uptake of colorectal cancer engagement, but engagement with many other elements of health promotion. Yet, despite this being reiterated in numerous pieces of literature and within communities, this is rarely addressed outright within research, health education materials, or operations. This lack of focus to date on the element of ‘mistrust’ is potentially due to the sensitivity surrounding the historical and ongoing structural issues that continue to affect tribal communities (i.e., colonization and historical trauma). However, without directly working through, investigating, and addressing mistrust head on, which is likely itself rooted in colonization, experiences of racism, white supremacy, and de-valuing of Indigenous ways of knowing and healing by Western medicine [11, 14, 15], health education initiatives may continue to butt up against a brick wall while disparities continue to be prevalent. Transformative ways of thinking about barriers and facilitators are needed.

Systems-thinking approaches [46] may offer a helpful way of elucidating relational aspects of shared barriers and facilitators leading to pathways for future research need. Systems-thinking has been defined as “an iterative learning process in which we replace a reductionist, narrow, short-run, static view of the world with a holistic, broad, long-term, [and] dynamic view” [47]. Systems-thinking approaches may be more in line with Indigenous ways of knowing due to the focus on circular thinking, being primarily relationship-based, and the ability to see health issues through an ‘eagle’s eye view’ [48, 49]. With this, more action-based research is needed to better clarify the *relationships* between the barriers and facilitators we identified such as that between various colorectal screening barriers and facilitating community level promotion activities. In addition, there is need for more in-depth study of the socio-cultural elements (e.g., colorectal cancer not talked about) within AI communities and how those elements can be considered and implemented directly within colorectal cancer education and promotion strategies through a strengths-based lens.

## Limitations

As noted previously, qualitative research in Indigenous communities allows for robust responses rooted in deep community understanding; however, qualitative approaches are considered limited in their ability to generalize research results. By comparing our results to research in other regions, we were, however, able to see consistency despite the research methods we used, giving us some confidence on the potential for transferability at a meta-level (still making note of the need to consider cultural nuances at an implementation level). Although we made a concerted effort to sample a wide range of tribal communities, ages, and sexes within the region, we may still have had a final study sample that was not representative of the broader tribal communities in the region. Due to saturation occurring within our data set, and the similarities in the results with existing research in other regions, we felt confident that our results contained relevant elements of consideration for North Dakota tribal communities.

## Conclusion

We sought to identify the perceived barriers and facilitators for the engagement with colorectal cancer prevention within North Dakota tribal communities. By working with communities, we were able to identify four main themes that were barriers for the engagement with colorectal cancer prevention in the region, including: colorectal cancer screening barriers, focus on other health problems, lack of colorectal cancer tailored health promotion, and socio-cultural factors affecting colorectal cancer prevention. We also identified three main themes that were facilitators for the engagement with colorectal cancer prevention in the region, including: reasons for getting colorectal cancer screening, role of culture, and getting out into the community. Ultimately, there is need for more community-rooted, strengths-based approaches to colorectal cancer prevention activities in AI communities in North Dakota that build off existing successes. Socio-cultural factors have been demonstrated to be an important element of consideration for colorectal cancer tribal community engagement and prevention planning in the state.

## Supplementary Information

**Additional file 1.**

## Data Availability

Due to ethical considerations and tribal data sovereignty, any data and materials associated with this research will not be available unless additional relevant tribal ethics agreements are in place.
