# Supplementary material for: Colorectal cancer community engagement: a qualitative exploration of American Indian voices from North Dakota
Source: BMC Cancer. 2022 Feb 9;22:158. doi: 10.1186/s12885-021-09119-2 (PMC8827199; doi:10.1186/s12885-021-09119-2)
Supplement: Supplementary file 1 — Additional file 1. [file 12885_2021_9119_MOESM1_ESM.pdf]

Interview Guide

Research Title: Colorectal cancer community engagement: A qualitative exploration of American Indian voices from North Dakota

-What do you think are the biggest strengths in your communities' approach to cancer prevention?

-What do you think are some of the biggest challenges in your communities' approach to cancer prevention?

-Do you think the number of cancer diagnoses has increased or decreased in the community in the last 5 years? Why do you think it might be increasing or decreasing?

-Do you think people from your community or region trust the information they hear about cancer from the health professionals and/or health system? Why do you think that may or may not be?

-Do you think cancer prevention related education materials need to be culturally and geographically adapted to your region? Why is this not the case? Why do you think this is important? Can you give an example of how materials might need to be adapted to your culture or region?

-What do you think your preferred method might be to learn about cancer prevention in your community? For example, is it easier to learn from reading brochures, or listening to others speak, or watching videos, or do you have other types of methods you prefer instead of these examples? Why do you think this way [preferred method] is easier to learn about cancer prevention than other types of learning methods?

-What types of resources are you aware of regarding colorectal cancer screening in your community?

-What are the biggest factors that impact you or your families' decision whether or not to get a colorectal cancer screen?

-Are there certain barriers to accessing colorectal cancer screening that you can think of in your community that it is important for the health system to be aware of?

-Do you think that there is a certain type of education needed around colorectal cancer screening in your community that is not currently being done?

-Do you see any role for traditional Indigenous knowledge, healing, and/or culture in promoting colon cancer screening in your community? Why or why not might this be the case?

-If you could see something improved right away in your community or regions approach to colorectal cancer prevention work, what would it be?
